# Interferon inhibits the release of herpes simplex virus-1 from the axons of sensory neurons

**DOI:** 10.1128/mbio.01818-23

**Published:** 2023-09-01

**Authors:** Kevin Danastas, Gerry Guo, Jessica Merjane, Nathan Hong, Ava Larsen, Monica Miranda-Saksena, Anthony L. Cunningham

**Affiliations:** 1 Centre for Virus Research, The Westmead Institute for Medical Research, Westmead, NSW, Australia; 2 Faculty of Medicine and Health, The University of Sydney, Westmead, NSW, Australia; University of Calgary, Calgary, Canada; Harvard Medical School, Boston, Massachusetts, USA

**Keywords:** herpes simplex virus, interferons, neurons, axons

## Abstract

Herpes simplex virus-1 (HSV-1) has evolved mechanisms to evade the host immune system and limit the antiviral effects induced by interferon (IFN) produced by local epithelial and immune cells. In this study, we determined the effects of type I, II, and III IFNs on HSV-1 release from sensory axons. Using compartmentalized microfluidic devices separating axons from the neuronal cell bodies, we showed that treating axons with type I (IFNα and IFNβ), type II (IFNγ), and type III (IFNλ) IFNs inhibited the release of HSV-1 from axons, without altering the anterograde axonal transport of viral components. Furthermore, we showed that only type II IFN induced a cell-wide response following axonal treatment, with phosphorylated STAT1 and STAT3 present along axons and in the neuronal cell body, whereas type I and III IFNs only induced a local STAT1 and STAT3 response in axons. We also showed that HSV-1 infection alone, in the absence of exogenous IFN, activated STAT1 and STAT3 in both neuronal cell bodies and along axons. However, HSV-1 infection restricted the translocation of pSTAT1 and pSTAT3 to the nucleus even in the presence of IFNs, suggesting viral evasion mechanisms are involved in limiting the IFN response in the neuronal cell body. Overall, our study indicates a key and greater antiviral role of IFNs in inhibiting HSV-1 release from axon termini than in the ganglia during recurrent infections and identifies a novel intervention site, the neuro-epidermal junction, for the development of new immunotherapies.

**IMPORTANCE:**

Herpes simplex virus-1 (HSV-1) is a human pathogen known to cause cold sores and genital herpes. HSV-1 establishes lifelong infections in our sensory neurons, with no cure or vaccine available. HSV-1 can reactivate sporadically and travel back along sensory nerves, where it can form lesions in the oral and genital mucosa, eye, and skin, or be shed asymptomatically. New treatment options are needed as resistance is emerging to current antiviral therapies. Here, we show that interferons (IFNs) are capable of blocking virus release from nerve endings, potentially stopping HSV-1 transmission into the skin. Furthermore, we show that IFNγ has the potential to have widespread antiviral effects in the neuron and may have additional effects on HSV-1 reactivation. Together, this study identifies new targets for the development of immunotherapies to stop the spread of HSV-1 from the nerves into the skin.

## INTRODUCTION

Herpes simplex viruses (HSV) (1 and 2) are members of the alphaherpesvirus family capable of establishing lifelong infections in sensory neurons of the peripheral nervous system. Following primary infection in the epithelium or mucosa, the virus infects the axon endings of innervating sensory nerves of the dorsal root ganglia (DRG) or trigeminal ganglia (TG). The virus undergoes retrograde axonal transport to the cell body where the viral genome is deposited into the nucleus, establishing a latent infection and evading the host immune system (1, 2). HSV can undergo periodic reactivation where the virus replicates in the neuronal cell body and undergoes anterograde axonal transport back to the original site of infection, forming herpetic lesions, or is shed asymptomatically (1, 2).

In the epithelium, the host immune system restricts viral replication and spread. Recurrent herpes lesions contain high levels of IFNs, which are involved in controlling HSV-1 levels and clearing the virus from this site (3). Infected keratinocytes produce type I IFNs (including IFNα, IFNβ, IFNκ, and IFNϵ), and plasmacytoid dendritic cells (DCs) infiltrating the upper dermis secrete high levels of IFNα (4 – 8). This early type I IFN response is vital in controlling HSV-1 in the early stages of replication (9 – 11). As the infection progresses, infiltrating CD4^+^ and CD8^+^ T cells are recruited to the site of infection producing type II IFN (IFNγ), as well as sentinel CD4^+^ and CD8^+^ resident memory T cells (TRMs) (12 – 17). A subset of these CD8^+^ T cells persist at the site of infection as TRMs for several months and are involved in immune surveillance to control the next HSV-1 reactivation (15, 18, 19). The role of type III IFNs during HSV-1 infection is less known; however, both epithelial and non-epithelial cells produce IFNλ upon HSV-1 and HSV-2 infection *in vitro* (20 – 22), suggesting that they may also be present in herpes lesions.

Upon secretion, IFNs will bind their respective receptors, type I to IFN alpha receptor (IFNAR), type II to IFN gamma receptor (IFNGR), and type III to IFN lambda receptor (IFNLR) (9). This activates downstream signaling pathways via the phosphorylation of signal transducer and activator of transcription (STAT) proteins, which translocate to the nucleus to induce the transcription of IFN stimulated genes (ISGs), establishing an antiviral state (9, 23 – 26). This commonly occurs through the canonical STAT1 signaling pathway, but can also signal through non-canonical pathways, such as through STAT3 (9, 26).

Our group has previously shown that the addition of IFNα and IFNγ to human skin explants resulted in decreased viral replication and plaque formation following transmission from axons (27). However, little research has looked into the effects of IFNs directly on axons and the effects on virus release.

We have used a compartmentalized neuronal culture system to separate axons from their cell bodies (28) to determine the effects of axonal IFNs treatment on HSV-1 release. Here, we show that direct treatment of axons with type I (α-1, α-14, and β), type II (γ), and type III (λ-3) IFNs results in a marked and significant inhibition of virus release from axons, without affecting the transport of key viral proteins along axons to the axon termini. We further show that STAT1 and STAT3, and their tyrosine phosphorylated forms pSTAT1 (tyr701) and pSTAT3 (tyr705) (representatives of canonical and non-canonical IFN signaling, respectively) are activated locally in axons following axonal treatment with type I, II, and III IFNs, but only type II IFN induces a cell-wide response, with pSTAT1 and pSTAT3 present in the nucleus following axonal IFNγ treatment. We also show that HSV-1 infection alone is also capable of inducing pSTAT1 and pSTAT3 activation; however, it also restricts the subsequent translocation of pSTAT1 and pSTAT3 to the nucleus even in the presence of IFNs. These findings highlight the key antiviral role of IFNs at the axon or nerve termini and viral evasion mechanisms employed by HSV-1 in limiting the IFN response in the neuronal cell body.

## MATERIALS AND METHODS

### Cells and viruses

HSV-1 GFP-pUS9 (Fg9) was provided by Renato Brandimarti (University of Bologna, Bologna, Italy) (29). HSV-1 stocks were passaged in Vero cells grown in Dulbecco’s modified Eagle’s medium (DMEM; Invitrogen, USA) supplemented with 9% fetal bovine serum (FBS) (Sigma, USA).

### Antibodies

Rabbit antibody against purified HSV-1 nuclear C capsids (PTNC) was kindly provided by Frazer Rixon (MRC Virology Unit, Institute of Virology, UK) (30). Mouse monoclonal anti-STAT1 (AHO0832) and rabbit polyclonal anti-phospho-STAT1 (Tyr701; 44–376G) and Hoescht solution were obtained from Thermo Fisher Scientific, USA. Mouse monoclonal anti-STAT3 (ab119352) and rabbit monoclonal anti-phospho-STAT3 (Tyr705; ab76315) were obtained from Abcam, USA. Alexa Fluor-labeled secondary antibodies were obtained from Thermo Fisher Scientific.

### Interferons

The following recombinant IFNs were used in this study: recombinant rat IFNα-1 (100 ng/mL), recombinant rat IFNα-14 (100 ng/mL), and recombinant rat IFNβ (500 U/mL) were obtained from PBL Assay Science, USA. Recombinant mouse IFNλ-3 (100 ng/mL) was obtained from R&D Systems, USA. Recombinant rat IFNγ (1,000 U/mL) was obtained from Peprotech, USA.

### Preparation of neuronal cultures in microfluidic devices

Dissociated DRG neurons from 6- to 7-day-old Wistar rat neonates were prepared as previously described (28, 31). Dissociated neurons were plated into microfluidic devices (SND450; Xona Microfluidics, USA) bonded to cover glasses as previously described (28, 31, 32). Neurons were grown in neurobasal medium supplemented with L-glutamine (4 mM; Invitrogen), B-27 supplement (Life Technologies), brain-derived neurotrophic factor (BDNF; 5 ng/mL; Sigma), and 7S nerve growth factor (100 ng/mL; Sigma) for 3 days at 37°C with 5% CO_2_ to allow the axons to grow into the axonal compartment of the device prior to HSV-1 infection.

### HSV-1 infection and IFN treatment of neurons

IFN was added to either the cell body or axonal compartment at the indicated concentrations either 24 h prior to infection (for IFNβ, IFNγ, and IFNλ-3) or at the same time as infection (for IFNα-1 and IFNα-14). IFN treatment was maintained for the duration of the experiment. HSV-1 GFP-pUS9 (1.7 × 10^6^ PFU in 350 µL) was added to the cell body compartment only, to infect the neurons for 2 h followed by 2× washes with fresh neurobasal medium. For confocal microscopy studies, to label neurons with axons penetrating into the axonal compartment, lipophilic tracer DiD (Invitrogen) was added to the axonal compartment (3 µL/mL) at the same time as HSV-1 infection of the cell body compartment. At 30 h post-infection (hpi), the media was collected from both the cell body and axonal compartments and stored at −80°C. Cultures were then fixed in 3% formaldehyde for 30 min at room temperature followed by the addition of 0.1% Triton X-100 for 4 min.

For studies of neuronal lysates, HSV-1 infection and IFN treatment were performed as above. At 24 and 30 hpi, the cell body and axonal compartments were lysed separately using the Isolate II Genomic DNA Kit (Bioline, UK).

Control experiments were performed to check for virus leakage between compartments during virus infection. HSV-1 was added to the cell body compartment in the absence of neurons and incubated at 37°C. At 30 hpi, the media was collected from both the cell body and axonal compartments to detect possible virus leakage (Fig. S1A). Additionally, control experiments were performed to check for IFN leakage between compartments during IFN treatment. IFNγ was added to the axonal compartment in the absence of neurons and incubated at 37°C. At 30 h, the media was collected from both the cell body and axonal compartments and IFNγ levels were measured using the rat IFNγ SimpleStep ELISA Kit (Abcam, USA) to detect possible IFN leakage (Fig. S1B). These controls were performed in conjunction with mock-infected controls, where neurons in the cell body compartments were mock infected.

### Droplet digital PCR (ddPCR)

Viral DNA from the culture media or cell lysates was extracted using the Isolate II Genomic DNA Kit (Bioline, UK), as per the manufacturer’s instructions. Primers for ddPCR were obtained from Sigma Aldrich. Primer pairs (5′-ATCAACTTCGACTGGCCCTT-3′ and 5′-CCGTACATGTCGATGTTCAC-3′) directed against HSV-1 gD gene produce a 179-bp product (33). ddPCR was performed to measure the levels of HSV-1 gD copy number present in the culture media or cell lysates as previously described (28). Each sample was run in duplicate with mock-infected controls and no template controls included in each run. All samples had a minimum of 10,000 accepted droplets.

### Fluorescent focus assays (FFA)

Fluorescent focus assays were prepared using culture media from each compartment as previously described (28). Cells were imaged on an Olympus VS120 Slidescanner microscope (Olympus, Japan) and fluorescent plaques counted using FIJI imaging software (34).

### Immunofluorescence and confocal microscopy

The cultures in compartmentalized microfluidic devices were immunostained *in situ* by addition of primary antibodies to both cell body and axonal compartments as previously described (28). Cultures were imaged using a Leica SP5 II confocal microscope using the Leica HyD hybrid detector (Leica Microsystems, Germany). Following acquisition, deconvolution was performed using Huygens Professional Software (SVI, The Netherlands). Quantitation of pSTAT1 and pSTAT3 nuclear staining was performed using FIJI imaging software. Images of pSTAT1 and pSTAT3 staining were converted to binary images and the percentage of positive (black) pixels against total area was determined for each cell nucleus. Hoescht solution was used to identify cell nuclei.

### Statistical analysis

Statistical analysis was performed using GraphPad Prism 8 (GraphPad, USA). Statistical tests used for each data set are specified in the relevant figure legends. A two-sided *P* < 0.05 was considered significant.

## RESULTS

### Virus release from axons is significantly reduced following axonal treatment with type I, II, and III IFNs

In this study, we used primary rat DRG neurons grown in compartmentalized microfluidic devices to determine the role of IFNs on virus release from axons. Dissociated neurons were seeded into the cell body compartment of the devices and grown for 3 days to allow axons to grow through the microgrooves into the axonal compartment (28). The axons in the axonal compartment were either pre-treated with IFN (IFNβ, γ, and λ-3) for 24 h or IFN (IFNα-1 and -14) was added to the axonal compartment at the same time of HSV-1 infection of the cell body compartment. Pilot studies indicated that IFNα was the only treatment capable of inhibiting virus release when applied at the same time as infection. Culture media from both compartments were collected at 30 hpi and processed for ddPCR and FFA.

There are 14 known subtypes of IFNα, with varying degrees of potency and receptor affinity (35). In this study, IFNα-1 and IFNα-14 were chosen as representatives of IFNα subtypes displaying low and high binding to IFNAR, and therefore low and high potency, respectively (35). While there are four subtypes of IFNλ in humans, only the λ-2 and λ-3 subtypes exist in rodents (36). Despite there being a 96% similarity between IFNλ-2 and IFNλ-3, IFNλ-3 is 16-fold more potent than IFNλ-2. Therefore, IFNλ-3 was chosen for this study (37).

**Fig 1:**
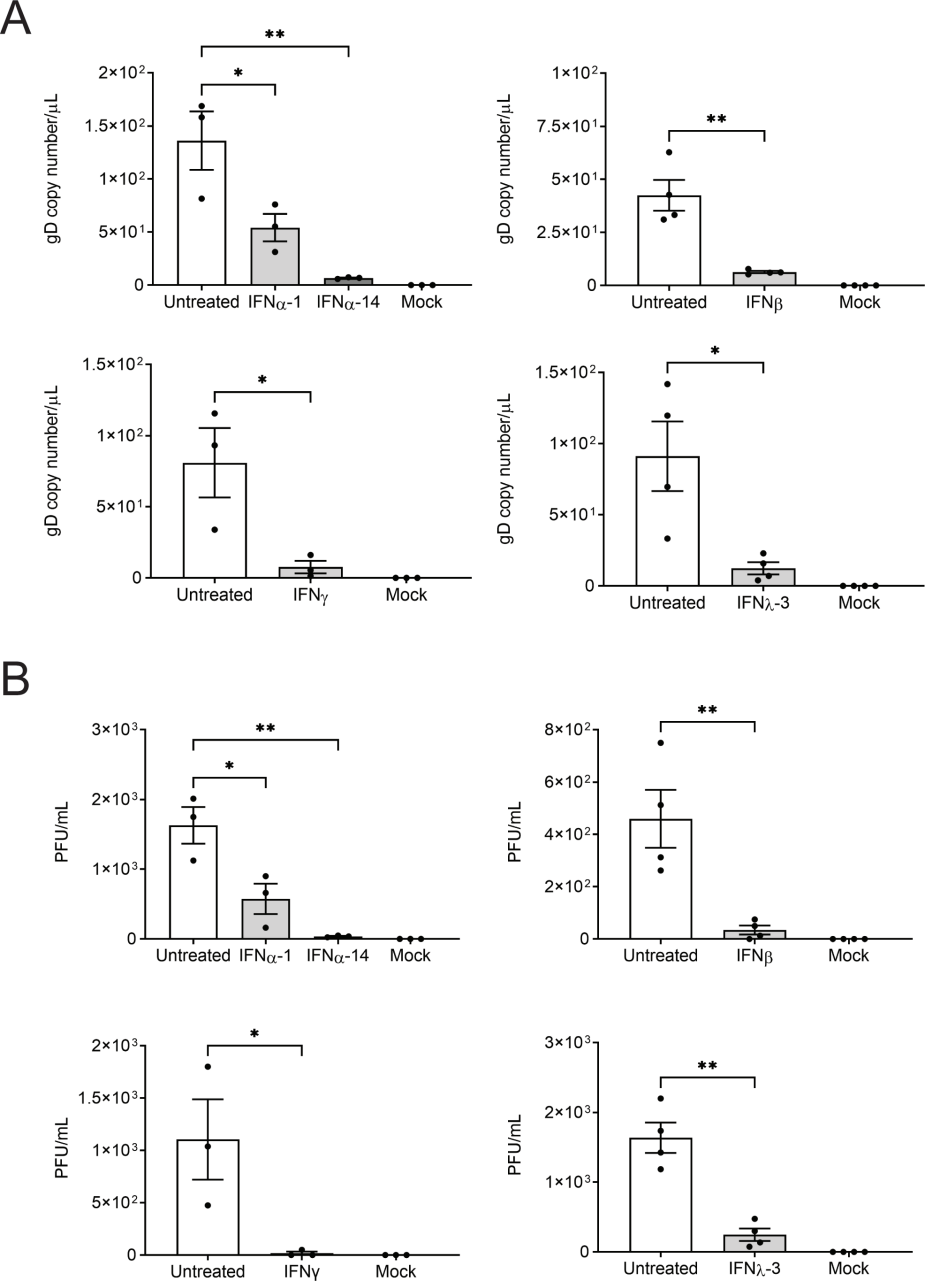
Axonal IFN treatment inhibits the release of HSV-1 from axons. Neonatal rat DRG neurons were dissociated and seeded into the cell body compartment of microfluidic devices. Cultures were grown for 3 days to allow axons to grow into the axonal compartment. The axonal compartments were pre-treated for 24 h with IFNβ (500 U/mL), IFNγ (1,000 U/mL), or IFNλ-3 (100 ng/mL) or at the same time as infection with IFNα-1 (100 ng/mL) or IFNα-14 (100 ng/mL). The cell body compartment was infected with HSV-1 and media collected from both compartments at 30 hpi. Viral DNA was extracted from the media and analyzed by ddPCR to measure the copy number of DNA encoding for viral envelope protein, glycoprotein D (gD). Infectious viral titres were also determined by FFA. Quantitation of viral release from the axonal compartment is shown following IFN treatment by ddPCR (**A**) and FFA (**B**). Statistical analysis was performed using a one-way analysis of variance (ANOVA) with Tukey’s *post hoc* analysis (for IFNα subtypes), or an independent student’s *t*-test (for IFNβ, γ, and λ-3) (*P < 0.05, **P < 0.01, ***P < 0.001, and ****P < 0.0001). Error bars represent the standard error of the mean (SEM). *n* = 3 for IFNα and IFNγ, and *n* = 4 for IFNβ and IFNλ-3.

A significant reduction in virus release in the media in the axonal compartment was observed by ddPCR following treatment with all IFNs tested compared to untreated infected controls [*P* < 0.05 (IFNα-1 and γ), *P* < 0.01 (IFNα-14, β, and λ-3), Fig. 1A]. The highest levels of inhibition were observed with IFNα-14 and IFNγ (95% ± 0.5% and 90% ± 8%, respectively), followed by IFNβ (85% ± 2%) and IFNλ-3 (82% ± 7%). IFNα-1 displayed the weakest level of inhibition (60% ± 13%).

Similar results were obtained by measuring infectious titre by FFA [*P* < 0.05 (IFNα-1 and γ), *P* < 0.01 (IFNα-14, β and λ-3), Fig. 1B]. IFNα-14 and IFNγ showed the highest levels of inhibition (98% ± 0.6% and 99% ± 2%, respectively), followed by IFNβ (93% ± 7%), IFNλ-3 (78% ± 14%), and IFNα-1 (65% ± 19%).

**Fig 2:**
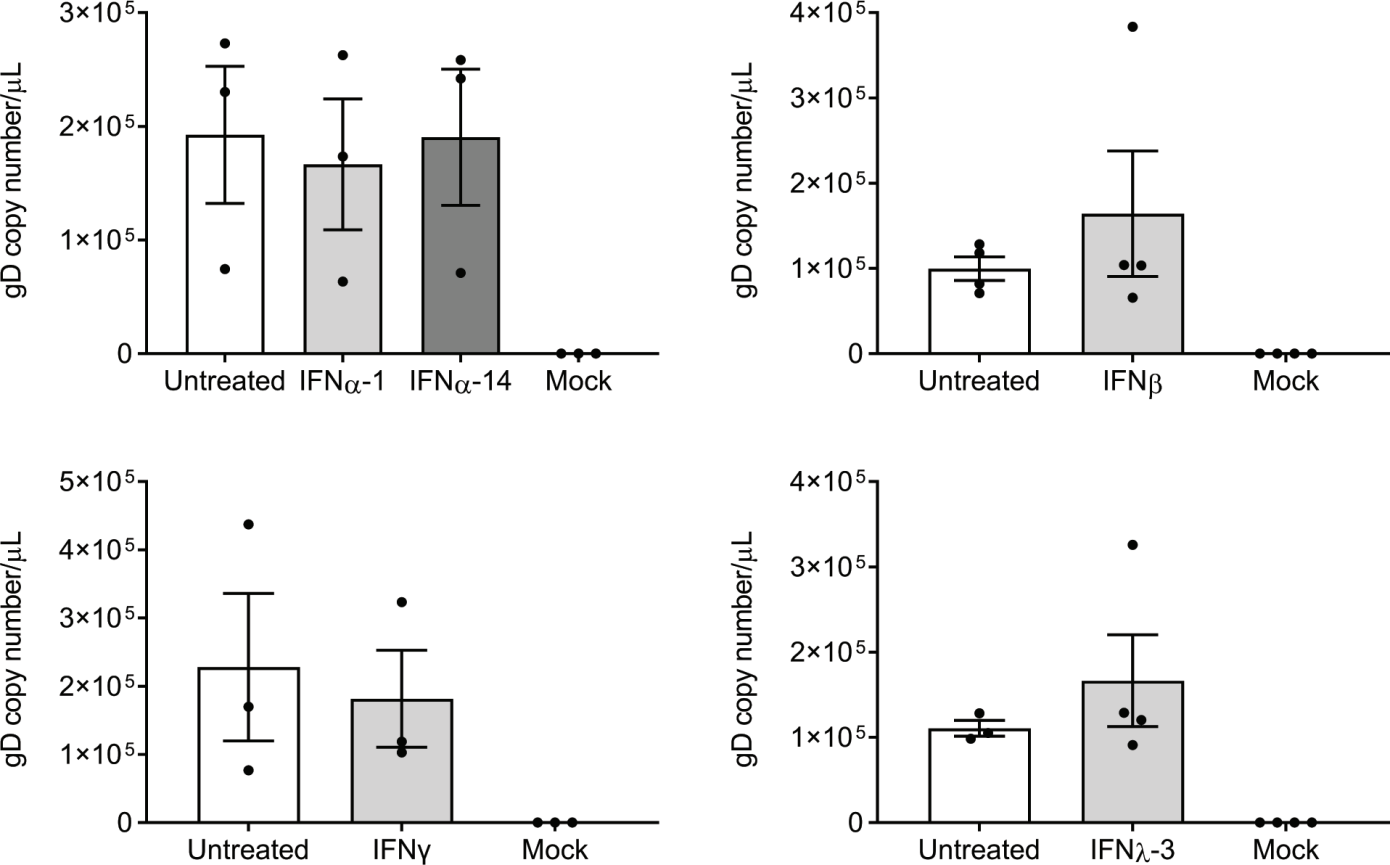
Axonal IFN treatment does not inhibit the release of HSV-1 from neurons in the cell body compartment. Neonatal rat DRG neurons grown in microfluidic devices were treated with IFN in the axonal compartment 24 h prior to infection (IFNβ, IFNγ, and IFNλ-3) or at the same time of infection (IFNα-1 and IFNα-14). The cell body compartment was infected with HSV-1 and media collected from both compartments at 30 hpi. Viral DNA was extracted from the media and analyzed by ddPCR to measure the copy number of DNA encoding for viral envelope protein, gD. Quantitation of viral release from the cell body compartment is shown following IFN treatment. *n* = 3 for IFNα and IFNγ, and *n* = 4 for IFNβ and IFNλ-3.

We have previously shown that ddPCR can reliably measure HSV-1 levels in the media from infected neuronal cultures, and that ddPCR shows high correlation to infectious titre measured by FFA (28). Therefore, ddPCR was used to measure HSV-1 released in the cell body compartment following either direct or axonal IFN treatment. No significant changes in virus release from neurons in the cell body compartment were observed following axonal IFN treatment (Fig. 2). Direct treatment of neurons in the cell body compartment with the IFNγ and IFNα-14 (which inhibited greater than 90% of virus release from axons) also showed no significant change in virus release (Fig. S2).

We additionally measured the levels of viral DNA present in both the neuronal cell bodies and axons to determine at which level of the neuron IFNγ was exerting its anti-viral effect. Axons in the axonal compartment were pre-treated for 24 h with IFNγ followed by HSV-1 infection in the cell body compartment. Neurons in the cell body compartment and axons in the axonal compartment were then lysed at 24 and 30 hpi and viral DNA was extracted and processed for ddPCR.

**Fig 3:**
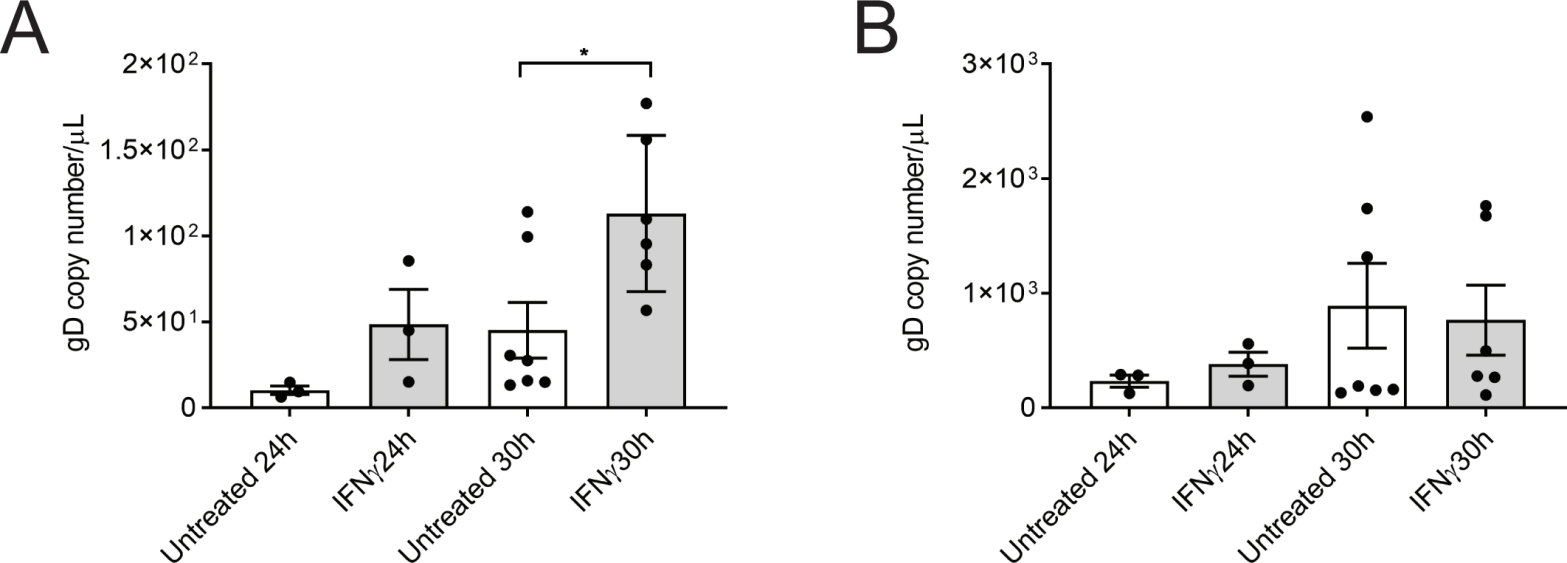
Axonal IFN treatment results in the accumulation of HSV-1 in axons. Neonatal rat DRG neurons grown in microfluidic devices were treated with IFNγ in the axonal compartment 24 h prior to infection. The cell body compartment was infected with HSV-1 and both compartments were lysed at 24 and 30 hpi. Viral DNA was extracted from cell and axon lysates and analyzed by ddPCR to measure the copy number of DNA encoding for viral envelope protein, gD. Quantitation of virus in the axonal (**A**) and cell body (**B**) compartments is shown following IFN treatment. Statistical analysis was performed using a one-way ANOVA with Tukey’s *post hoc* analysis (*P < 0.05). Error bars represent the SEM. *n* = 3 for 24 hpi and *n* = 6-7 for 30 hpi.

There was a significant increase in HSV-1 DNA level in axons treated with IFNγ at 30 hpi compared to untreated infected controls (*P* < 0.05, Fig. 3A). No significant changes in viral DNA levels were detected in axons, untreated or IFNγ treated, at 24 hpi (Fig. 3A) or in cell bodies at 24 or 30 hpi, following axonal IFNγ treatment (Fig. 3B).

### Axonal IFN treatment does not affect axonal transport of viral proteins from the cell body to axon termini

We next determined whether the replication of the virus and axonal transport of viral components along axons to axon termini was affected by axonal IFN treatment. The axonal compartments were treated with IFN as described above and the lipophilic tracer (DiD) was added to the axonal compartment at the same time of HSV-1 infection of the cell body compartment. At 30 hpi, cultures were fixed and processed for confocal microscopy. Neurons were stained for viral proteins and imaged using a Leica SP5 II confocal microscope.

**Fig 4:**
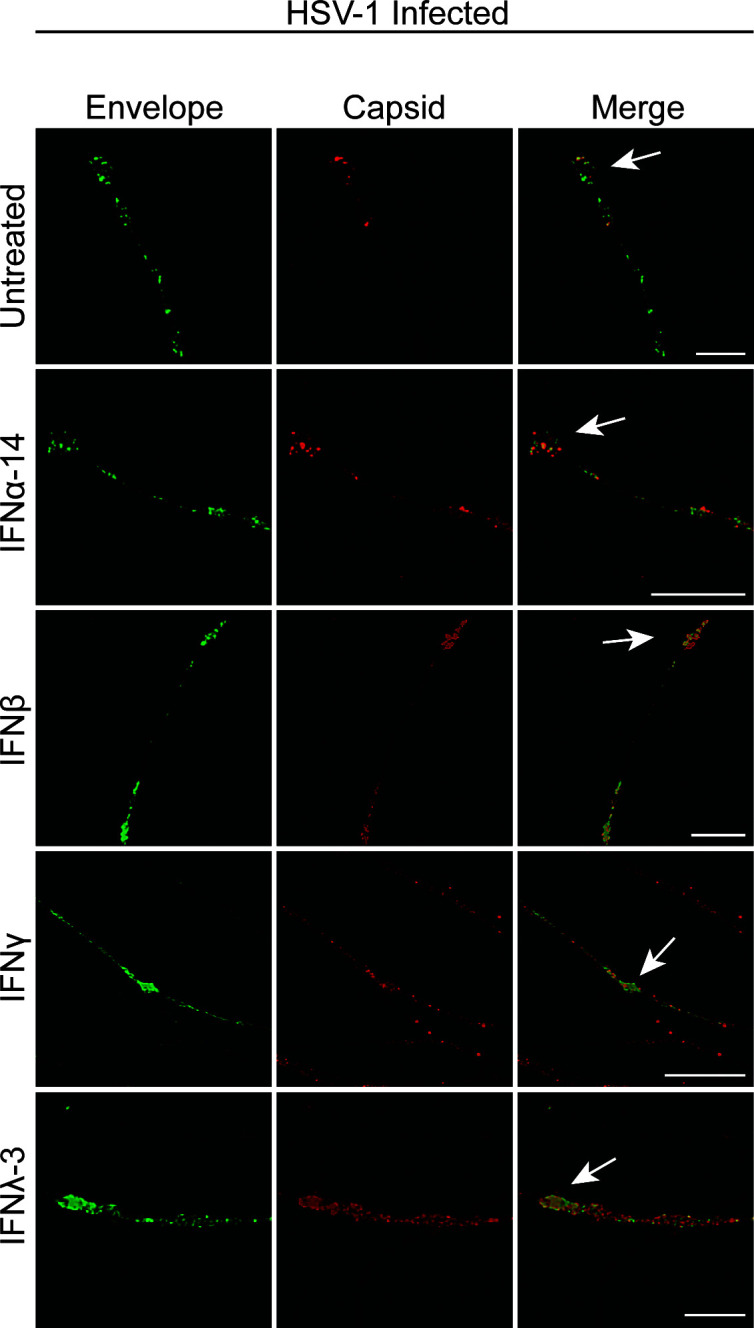
Axonal IFN treatment does not inhibit anterograde axonal transport of HSV-1 capsid and envelope proteins along axons. Axons in the axonal compartment were treated with IFN 24 h prior to infection (IFNβ, IFNγ, and IFNλ-3) or at the same time of infection (IFNα-14). Neurons in the cell body compartment were infected with HSV-1 and the cultures were fixed at 30 hpi. Cultures were immunostained for HSV-1 C capsids. Cultures were examined using a Leica SP5 II confocal microscope. Micrographs of HSV-1 infected axons showing label for viral envelope protein pUS9 (GFP-green) and viral capsid (red) following axonal treatment with IFNα-14, β, γ, or λ-3. Axon termini are indicated by arrows. Scale bars = 10 µm.

Label for viral envelope (pUS9) and capsid proteins was present along axons, in varicosities (located at axonal branch points) and in axon termini in all treatment conditions. No differences were observed in viral protein distribution following IFN treatment compared to the untreated infected control (Fig. 4). Colocalization between viral envelope and capsid proteins was evident in axonal varicosities and axon termini in all conditions (Fig. 4).

**Fig 5:**
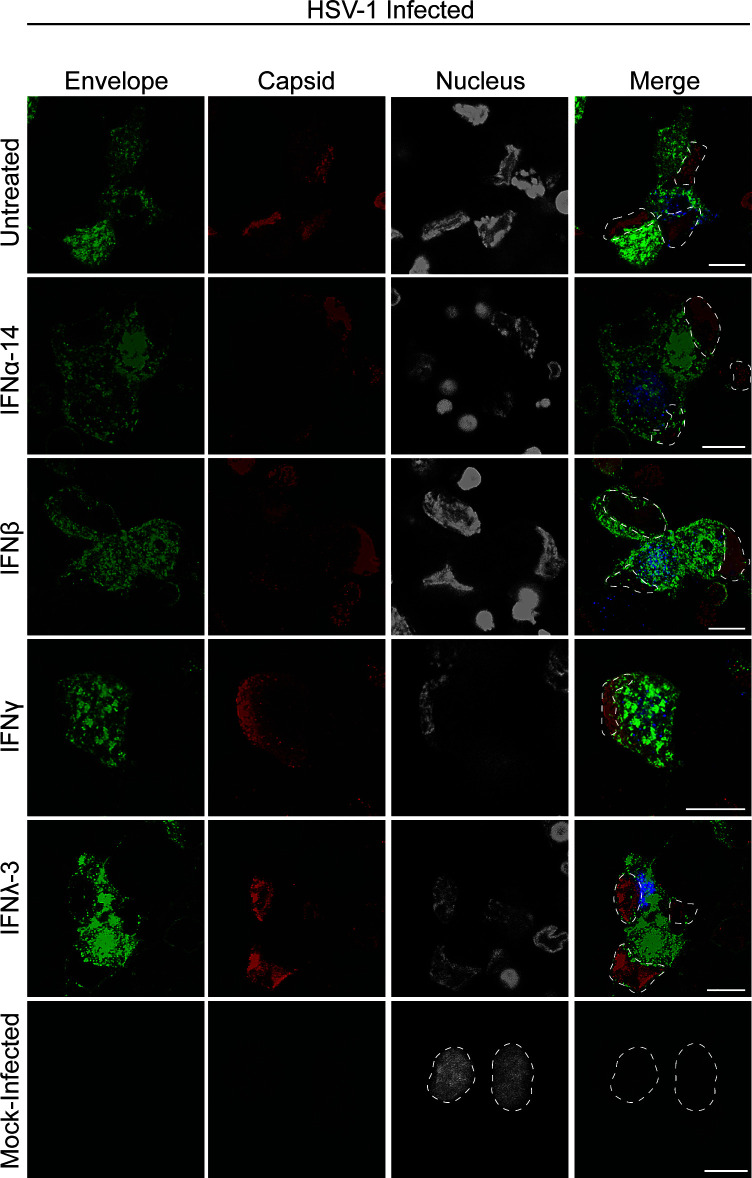
Axonal IFN treatment does not affect viral replication or viral protein distribution in the cytoplasm of the cell body. Axons in the axonal compartment were treated with IFN in the axonal compartment 24 h prior to infection (IFNβ, IFNγ, and IFNλ-3) or at the same time of infection (IFNα-14). Neurons in the cell body compartment were infected with HSV-1 or mock infected and the lipophilic tracer (DiD) was added to the axonal compartment. Cultures were fixed at 30 hpi and immunostained for HSV-1 C capsids. Cultures were examined using a Leica SP5 II confocal microscope. Micrographs of HSV-1 infected axons showing label for viral envelope protein pUS9 (green), viral capsid (red), nuclei (grey), and lipophilic tracer (blue) following axonal treatment with IFNα-14, β, γ, or λ-3. Dashed lines in the merged panel represent relevant nuclei. Scale bars = 10 µm.

The lipophilic tracer DiD was used to identify neuronal cell bodies with axons extending into the axonal compartment in both untreated and IFN-treated samples. The distribution of viral envelope (pUS9) and capsid proteins in the cell body was unaffected by axonal IFN treatment (Fig. 5). Consistent with previous findings (28), viral envelope (pUS9) was present diffusely in the cytoplasm and absent from the nucleus, whereas viral capsid proteins were concentrated in the nucleus with weak cytoplasmic staining (Fig. 5).

### STAT1 and STAT3 are activated in neurons by both IFN and HSV-1 infection alone but HSV-1 infection limits their nuclear translocation

We next investigated the signaling pathways that IFN activates in sensory neurons. We examined the effects of direct treatment with IFN on the neuronal cell bodies. Neurons in the cell body compartment of microfluidic devices were directly treated with IFNα-14, IFNβ, IFNγ, or IFNλ-3 for 24 h prior to HSV-1 or mock infection. Cultures were then fixed at 30 hpi, immunostained for STAT1, pSTAT1 (tyr701), STAT3, and pSTAT3 (tyr705) and imaged using a Leica SP5 II confocal microscope.

**Fig 6:**
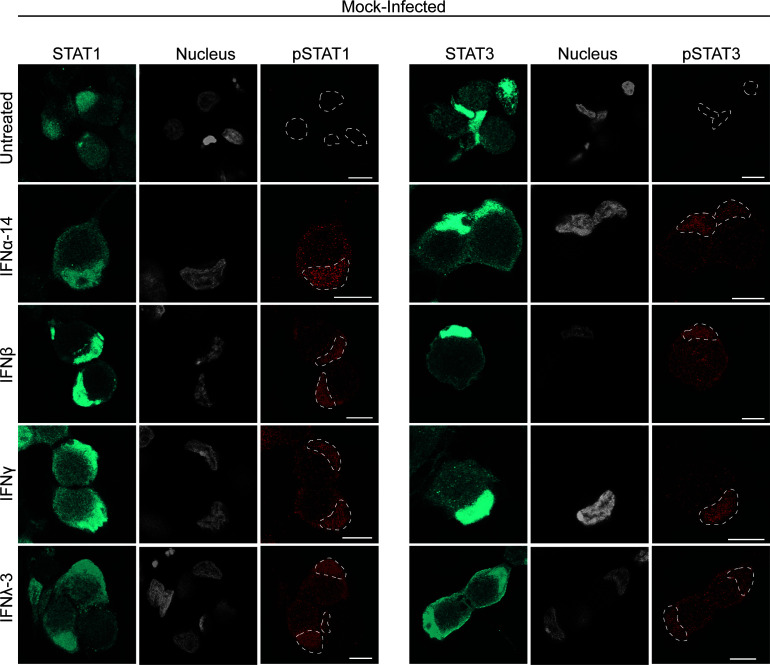
Type I, II, and III IFN treatment of neuronal cell bodies activates STAT1 and STAT3. Neurons in the cell body compartment were directly treated with IFN for 24 h prior to mock infection. Cultures were fixed at 30 hpi and immunostained for either STAT1 and pSTAT1 (tyr701), or STAT3 and pSTAT3 (tyr705). Cultures were examined using a Leica SP5 II confocal microscope. Micrographs of mock-infected cell bodies showing label for either STAT1 (cyan) and pSTAT1 (red), or STAT3 (cyan) and pSTAT3 (red), and nuclei (grey) following direct treatment with IFNα-14, β, γ, or λ-3. Dashed lines in the pSTAT1 and pSTAT3 panels represent relevant nuclei. Scale bars = 10 µm.

**Fig 7:**
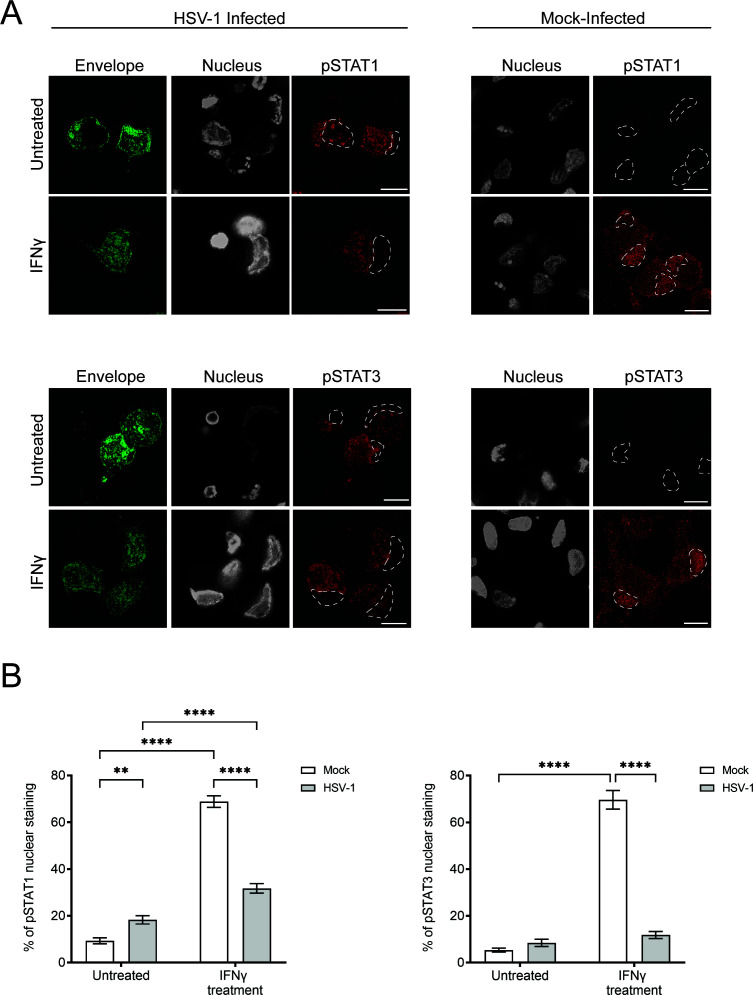
HSV-1 infection alone activates STAT1 and STAT3 in sensory neurons and limits the nuclear translocation of pSTAT1 and pSTAT3. Neurons in the cell body compartment were directly treated with IFNγ for 24 h prior to HSV-1 or mock infection. Cultures were fixed at 30 hpi and immunostained for either pSTAT1 (tyr701), or pSTAT3 (tyr705). Cultures were examined using a Leica SP5 II confocal microscope. (**A**) Micrographs of HSV-1 and mock-infected neuronal cell bodies showing label for viral envelope protein pUS9 (green), nuclei (grey), and either pSTAT1 (red) or pSTAT3 (red). Dashed lines in the pSTAT1 and pSTAT3 panels represent relevant nuclei. Scale bars = 10 µm. (**B**) Quantification of pSTAT1 and pSTAT3 nuclear staining in HSV-1 and mock-infected neurons with and without direct IFNγ pretreatment. Statistical analysis was performed using a two-way ANOVA with Tukey’s *post hoc* analysis (**P < 0.01, ****P < 0.0001). Error bars represent the SEM. *n* = 43–58 cells per condition.

Label for STAT1 and STAT3 was present diffusely throughout the cytoplasm and concentrated in the nucleus in mock-infected neuronal cell bodies when untreated or treated with IFN (Fig. 6). However, label for pSTAT1 and pSTAT3 was concentrated in the nucleus of IFN treated mock-infected neurons and absent in untreated mock-infected neurons (Fig. 6). Interestingly, during HSV-1 infection in the absence of IFN treatment, we also observed that both STAT1 and STAT3 were activated (Fig. 7A). Label for pSTAT1 and pSTAT3 was present in neuronal cell bodies of HSV-1 infected neurons, but in a different pattern of distribution compared to IFN treated mock-infected neurons. Label for pSTAT1 and pSTAT3 was mainly present in a punctate pattern throughout the cytoplasm of HSV-1 infected neurons with only weak staining in the nucleus (Fig. 7A). This staining pattern for pSTAT1 and pSTAT3 was similar in HSV-1 infected neurons that were also directly treated with IFNγ (Fig. 7A) and IFNα-14, β, and λ (Fig. S3). In HSV-1 infected cultures, label for pSTAT1 and pSTAT3 was only observed in neurons positive for HSV-1, identified by GFP-pUS9. Label for pSTAT1 and pSTAT3 was absent in adjacent uninfected neurons (negative for GFP-pUS9).

HSV-1 infection limited the translocation of pSTAT1 and pSTAT3 to the nucleus even in the presence of IFN (all subtypes) (Fig. 7A; see also Fig. S3). Therefore, we quantified the levels of pSTAT1 and pSTAT3 nuclear staining in HSV-1 and mock-infected neurons, with and without direct IFNγ treatment (Fig. 7B). Nuclear staining for both pSTAT1 and pSTAT3 was significantly higher in mock-infected neurons treated with IFNγ, compared to untreated mock-infected neurons (*P* < 0.0001, Fig. 7B). Unexpectedly, there was also a moderate but significant increase in pSTAT1, but not pSTAT3, nuclear staining in HSV-1 infected neurons compared to the baseline nuclear staining of mock-infected neurons (*P* < 0.01, Fig. 7B). Similarly, IFNγ treated HSV-1 infected neurons had significantly higher nuclear staining for pSTAT1 compared to untreated HSV-1 infected neurons (*P* < 0.0001, Fig. 7B). However, no difference in pSTAT3 nuclear staining was observed between IFNγ treated and untreated HSV-1 infected neurons. Most interestingly, IFNγ treated HSV-1 infected neurons had significantly less pSTAT1 and pSTAT3 nuclear staining compared to IFNγ treated mock-infected neurons (*P* < 0.0001, Fig. 7B), supporting our observations that HSV-1 infection limits nuclear translocation of pSTAT1 and pSTAT3 induced by IFN treatment (Fig. 7B).

### Axonal IFN treatment activates STAT1 and STAT3 locally in axons

We next investigated the activation of STAT1 and STAT3 in neurons following the addition of IFN to only the axonal compartment of both HSV-1 and mock-infected neurons. The axonal compartment was treated with IFN as described above and neurons in the cell body compartment were infected with HSV-1. The lipophilic tracer DiD was added to the axonal compartment at the same time as IFN treatment to identify neurons in the cell body compartment with axons extending into the axonal compartment and thus exposed to axonal IFN treatment. At 30 hpi, cultures were fixed and processed for confocal microscopy. Neurons were stained for STAT1, pSTAT1 (tyr701), STAT3, and pSTAT3 (tyr705) and imaged using a Leica SP5 II confocal microscope.

**Fig 8:**
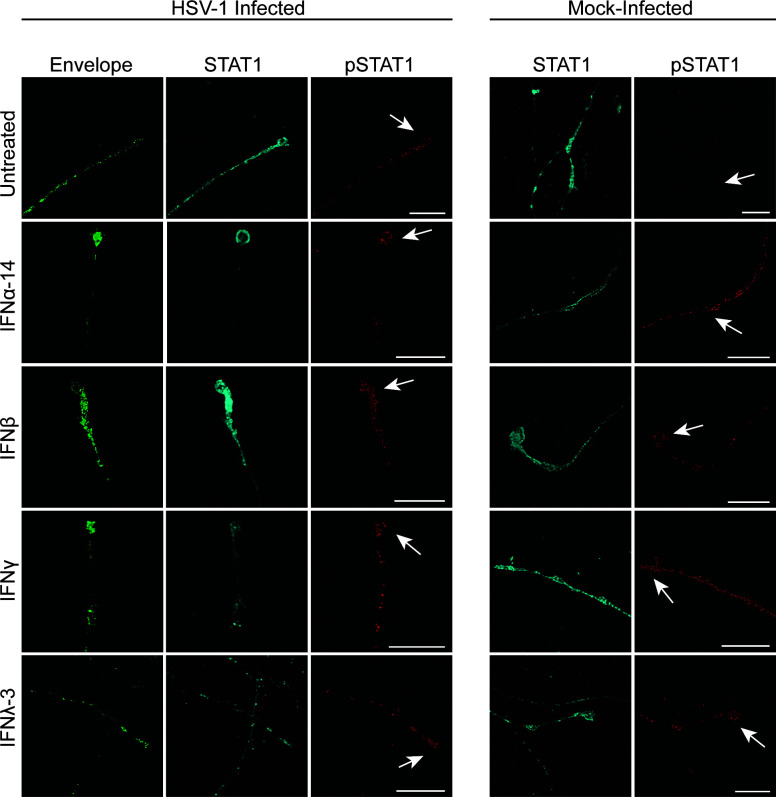
Axonal IFN treatment activates STAT1 locally in axons. Axons in the axonal compartment were treated with IFNα-14, IFNβ, IFNγ, or IFNλ-3 as previously described followed by HSV-1 or mock infection of the cell body compartment. Cultures were fixed at 30 hpi and immunostained for STAT1 and pSTAT1 (tyr701). Cultures were examined using a Leica SP5 II confocal microscope. Micrographs of HSV-1 or mock-infected axons showing label for viral envelope protein pUS9 (green), STAT1 (cyan), and pSTAT1 (red). Axon termini and varicosities are indicated by arrows. Scale bars = 10 µm.

**Fig 9:**
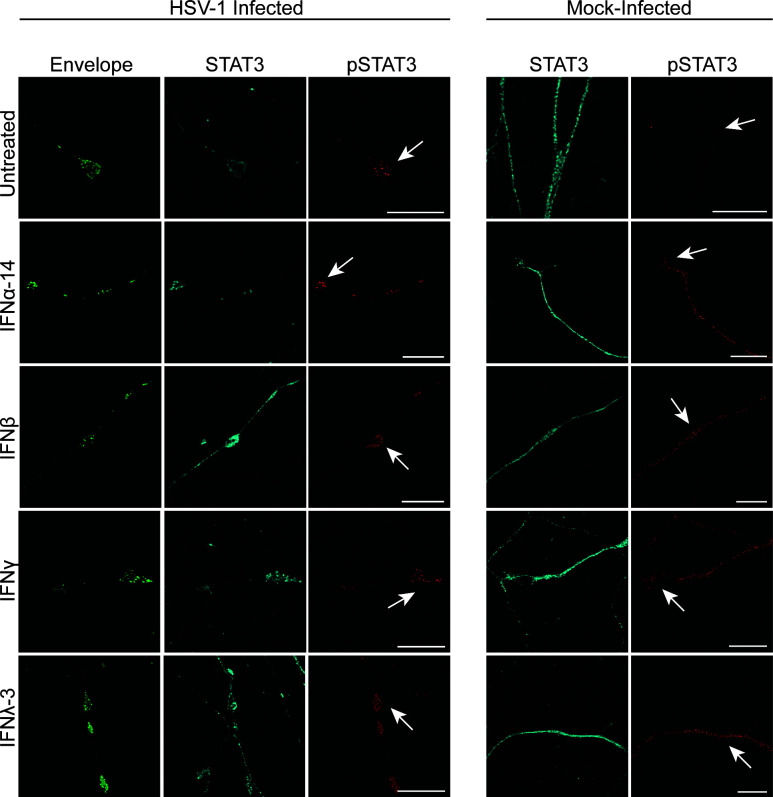
Axonal IFN treatment activates STAT3 locally in axons. Axons in the axonal compartment were treated with IFNα-14, IFNβ, IFNγ, or IFNλ-3 as previously described followed by HSV-1 or mock infection of neurons of the cell body compartment. Cultures were fixed at 30 hpi and immunostained for STAT3 and pSTAT3 (tyr705). Cultures were examined using a Leica SP5 II confocal microscope. Micrographs of HSV-1 or mock-infected axons showing label for viral envelope protein pUS9 (green), STAT3 (cyan), and pSTAT3 (red). Axon termini and varicosities are indicated by arrows. Scale bars = 10 µm.

Label for STAT1 and STAT3 was present diffusely along axons in both HSV-1 and mock-infected axons untreated or treated with IFNα-14, β, γ, or λ-3 (Fig. 8 and 9). Label for pSTAT1 and pSTAT3 was absent in mock (untreated) axons but was present along mock-infected axons treated with IFN as well as in HSV-1 infected axons, untreated or treated with IFN (Fig. 8 and 9).

Label for pSTAT1 and pSTAT3 was present in a punctate pattern along axons in the axonal compartment of HSV-1 infected neurons. The distribution of label for pSTAT1 and pSTAT3 along HSV-1 infected axons was similar to that of HSV-1 infected axons directly treated with IFN (Fig. 8 and 9). Adjacent uninfected (untreated) axons in the axonal compartment did not express pSTAT1 or pSTAT3.

### Only type II IFN induces nuclear translocation of pSTAT1 and pSTAT3 in the neuronal cell body of mock-infected neurons

In order to determine whether addition of IFN directly to axons induces STAT signaling back to the neuronal cell body and subsequent pSTAT nuclear translocation, label for pSTAT1 and pSTAT3 was examined in only mock-infected neurons in compartmentalized microfluidic devices following IFN treatment of the axonal compartment. This is because HSV-1 infection alone was observed to activate STAT in the neuronal cell body and to also limit STATs nuclear translocation (Fig. 7; see also Fig. S3). The lipophilic tracer DiD was added to the axonal compartment at the same time as IFN treatment to identify neurons with axons extending into the axonal compartment being exposed to axonal IFN treatment.

**Fig 10:**
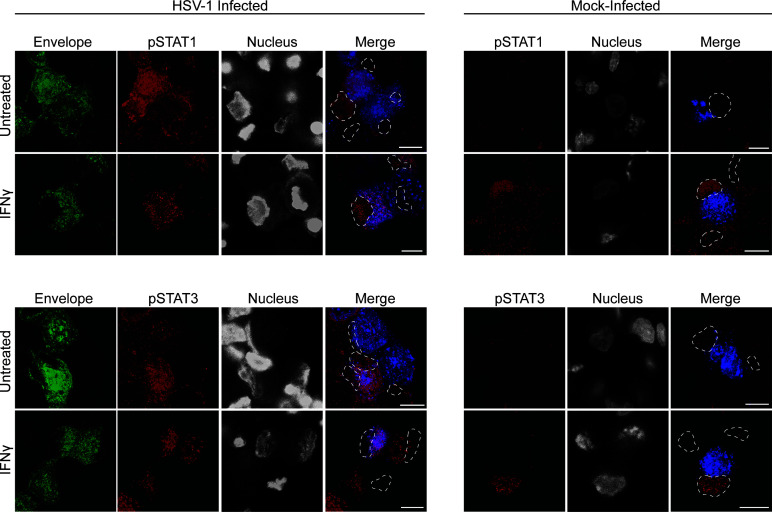
Axonal treatment with type II IFN results in a cell-wide response, inducing the nuclear translocation of pSTAT1 and pSTAT3 in mock-infected neurons. Axons in the axonal compartment were treated with IFNγ for 24 h prior to mock infection. The cell body compartment was infected with HSV-1 or mock infected and the lipophilic tracer (DiD) was added to the axonal compartment. Cultures were fixed at 30 hpi and immunostained for either pSTAT1 (tyr701) or pSTAT3 (tyr705). Cultures were examined using a Leica SP5 II confocal microscope. Micrographs of HSV-1 or mock-infected neurons showing label for viral envelope protein pUS9 (green), lipophilic tracer (blue), nuclei (grey), and either pSTAT1 (red) or pSTAT3 (red). Dashed lines in the merged panel represent relevant nuclei. Scale bars = 10 µm.

Only IFNγ treatment of axons in the axonal compartment resulted in concentration of pSTAT1 and pSTAT3 in the nucleus of the neuronal cell body of mock-infected neurons (which had axons extending into the axonal compartment) (Fig. 10; see also Fig. S4).

We also looked at additional timepoints to determine if nuclear pSTAT1 translocation occurred at an earlier timepoint following IFN treatment. Mock infected neurons were treated with IFNα-14 and IFNγ in the axonal compartment as above, in the presence of DiD. Cultures were fixed at 2, 18, and 30 h post treatment and stained for pSTAT1. Label for pSTAT1 was observed to be concentrated in the nucleus of the neuronal cell body and along axons of mock-infected neurons following axonal IFNγ treatment (Fig. S5). Label for pSTAT1 was present along axons but absent from the nucleus following axonal IFNα-14 treatment at all timepoints (Fig. S5).

## DISCUSSION

**Fig 11:**
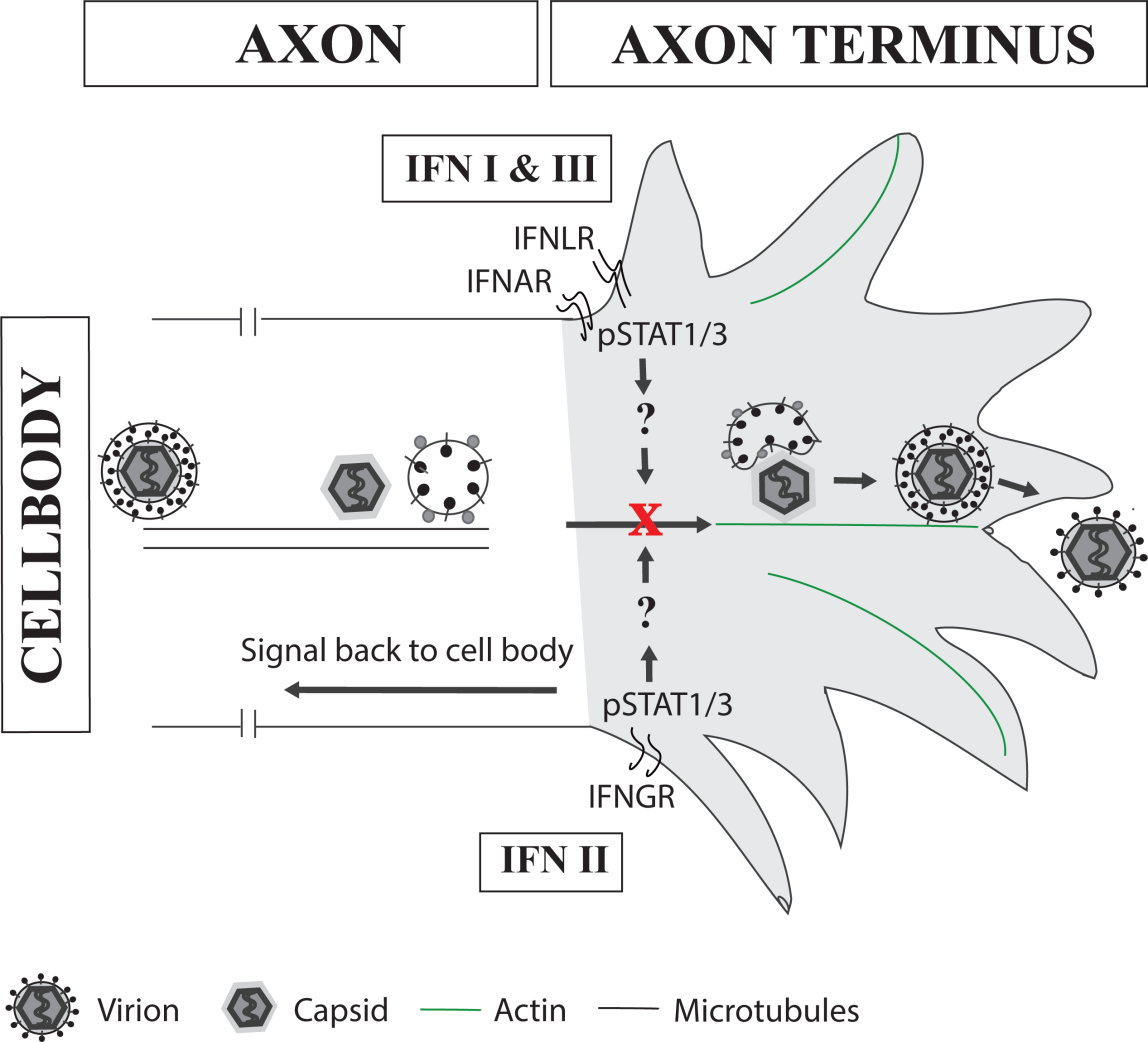
Schematic diagram of the inhibition of virus exit from axons by axonal IFN treatment. Type I, II, and III IFNs bind to their respective receptors on the axonal membrane and elicit a local response in the axon, signaling through pSTAT1 and/or pSTAT3 via an undefined signaling mechanism(s) to inhibit viral assembly, actin-mediated transport, or exocytosis. Type II IFN also has cell-wide effects, signaling to the cell body and inducing the nuclear translocation of pSTAT1/3.

Axonal IFN treatment inhibits retrograde axonal transport of HSV-1 towards the neuronal cell body (38, 39); however, the role of IFN in anterograde axonal transport, and subsequent assembly and exit of HSV-1 from axons remains unclear. In this study, we used a compartmentalized neuronal culture system to show that exposure of axons to type I (α-1, α-14, and β), II (γ), and III (λ-3) IFNs significantly inhibited virus release from axons. Importantly, this inhibition did not affect virus release from the neuronal cell body or anterograde axonal transport of viral components along axons to the axon termini (Fig. 11). All IFN treatments resulted in STAT1 and STAT3 phosphorylation locally in axons; however, only IFNγ induced such a response in the cell body (Fig. 11).

During recurrent herpes infections, HSV-1 reactivates in sensory neurons and following replication, undergoes anterograde axonal transport where the virus exits axon termini to infect the epidermis (1). Once in the epidermis, HSV-1 induces the production of types I and II IFNs from both infected epithelial cells as well as from resident and infiltrating immune cells, with high levels of types I and II IFNs present in the vesicle fluid obtained from recurrent lesions (3, 9, 40). The presence of IFN at this site is crucial in controlling viral replication in epidermal cells and for the clearance of virus from the lesion (9, 27, 41, 42). In particular, the secretion of IFNγ from resident memory CD8^+^ T cells in the upper dermis/epidermis after encountering recurring HSV has been modeled to prevent spread, control asymptomatic shedding, and reduce clinical lesion frequency (43, 44). We have previously investigated the inhibitory effects of IFNα and IFNγ on HSV-1 transmission using a dual chamber system containing human fetal DRGs innervating autologous skin explants (27). We showed that both IFNα and IFNγ reduced the size and number of infectious plaques in epidermal cells following axonal transmission of HSV-1. However, it was unclear whether this inhibitory effect was due to IFN acting only on the epidermal cells, or if the IFN exerted an inhibitory effect on the axons as well (27). Our current data support an additional role for IFN in the neuro-epidermal junction in controlling the actual release of HSV-1 from axon termini into the epidermis following anterograde transport, in addition to inhibiting viral replication in epidermal cells.

STAT1 is a key early signaling molecule in the canonical signaling pathways of type I, II, and III IFNs (9), activated immediately after IFN binds to their receptors. STAT1, STAT3, and STAT6 are the only STAT molecules that have been detected in the axoplasm of rat DRGs (45). Our data have shown that both STAT1 and STAT3 signaling pathways are activated in mock-infected axons following axonal treatment with type I, II, and III IFNs. Previous reports have shown that only IFNγ can induce a cell-wide response leading to the nuclear translocation of pSTAT1, whereas IFNβ only produces a localized pSTAT1 response in the axons (38, 39). Our work is consistent with these reports but also shows that pSTAT3 follows a similar pattern. Following axonal treatment with type I and III IFNs, both pSTAT1 and pSTAT3 were localized only to the axons and were absent from the neuronal cell body of mock-infected neurons. This observation supports the hypothesis that STAT1 and STAT3 in the axon are involved in non-canonical signaling pathways that are disrupting the viral lifecycle without inducing gene transcription in the nucleus (38, 39). Nevertheless, the long interval required for pretreating axons with IFNγ or even after the axonal addition of IFNα at the same time as HSV-1 infection in the cell body compartment, to obtain inhibition, suggests the need for downstream pathways in the axon, such as previously described pSTAT1 interaction with the mammalian target of rapamycin (mTOR) (39, 46 – 48). pSTAT1 can activate mTOR, which can be locally translated on axonal ribosomes and mediate protein translation in axons including STAT3 and other signaling molecules (49).

Following IFNα/β binding to their receptor IFNAR, or IFNλ binding its receptor IFNLR, canonical IFN signaling involves the formation of a STAT1–STAT2 heterodimer, which then forms a complex with IRF9, which together translocates to the nucleus for gene transcription (9, 50, 51). Canonical signaling for type II IFNs, however, follows a different pathway. Upon IFNγ binding its receptor IFNGR, STAT1 forms homodimers, which translocates to the nucleus for gene transcription (51). Given that only STAT1, 3, and 6 are present in the axoplasm of rat DRG neurons, and STAT2 is absent (45), this could potentially explain why canonical signaling is only observed following axonal treatment with type II IFNs, and not type I or III IFNs.

IFNγ is thought to play a significant role in the maintenance of HSV-1 latency and reactivation in the ganglia. HSV-1 specific CD8^+^ T cells cluster around neurons in HSV-1 latently infected TGs, with high levels of IFNγ present (52, 53), where it likely plays a role in limiting HSV-1 reactivation (54 – 56). Despite this, Sainz Jr. et al*.* showed that IFNγ and IFNβ are weak inhibitors of HSV-1 replication in several cell types, particularly in neurons (SK-N-SH cells), where only a twofold (IFNγ) and fivefold (IFNβ) inhibition was observed, compared to acyclovir which resulted in a 5,400-fold inhibition (57). Similarly, in this study, we show that direct treatment of the neurons with IFNγ only reduced HSV-1 release by 20% in the cell body compartment. In comparison, axonal treatment with IFNγ inhibited >90% of HSV-1 release from axons. Similarly, axonal treatment with IFNα-14 also inhibited >90% of HSV-1 release from axons but only inhibited 20% of HSV-1 release from neurons in the cell body compartment when neurons were directly treated with IFNα-14. The difference in inhibitory effects of IFN on the egress of HSV-1 from axons and the cell body of neurons is striking and unexpected. Liu et al. (56) reported that IFNγ can inhibit HSV-1 reactivation from latency of murine sensory neurons cultured *ex vivo* in the presence of acyclovir, but only immediately after acyclovir is removed, and not 24 h later. This finding that IFNγ cannot inhibit productive infection of rodent neurons at the level of the cell body is consistent with our data. Furthermore, our results would also help explain that after breakthrough of HSV-1 reactivation in the DRG or TG, the production of IFNs in the innervated epidermis, which can also act on nerve termini is critically important in controlling HSV-1 recurrences locally, whether symptomatic or asymptomatic, as indicated by the human studies mentioned above (43, 44).

Limited research is available on the effects of IFN on axonal transport, particularly anterograde axonal transport of HSV-1. Previous studies on HSV-1, and the closely related pseudorabies virus (PRV), have shown that retrograde axonal transport of viral capsid proteins, mediated by the microtubule motor protein dynein, is reduced following axonal IFN treatment (38, 39). The authors hypothesized that local axonal stimulation by pSTAT1 induces axonal protein synthesis and formation of retrograde injury signaling complexes that compete for the fast retrograde transport machinery mediated by dynein (39). Both retrograde and anterograde axonal transport of acidic organelles were unaffected by axonal IFN treatment, suggesting that IFN is specifically inhibiting retrograde transport of HSV-1 and PRV (39). They also suggest that pSTAT induced locally by IFN in the axon may act locally (e.g., by interacting with mTOR). However, our data show that the anterograde transport of HSV-1, which is mediated by kinesin motor proteins, is unaffected by IFN treatment and that the site of IFN action is quite different, probably on exocytosis, actin mediated transport or viral assembly in the axon terminus (Fig. 11).

In our study, we also observed that HSV-1 infection alone, in the absence of IFN treatment, was also capable of triggering the phosphorylation of STAT1 and STAT3. This was observed in both the axons and the neuronal cell body. However, surprisingly, in the presence of HSV-1, pSTAT1 and pSTAT3 were localized mostly in the cytoplasm with markedly reduced nuclear translocation. This distribution pattern was not altered by direct treatment of the neurons in the cell body compartment with any IFN prior to HSV-1 infection. In contrast, direct IFN treatment of mock-infected neurons in this compartment resulted in pSTAT1 and pSTAT3 concentrating in the nucleus, in line with expected IFN signaling (9). We further confirmed this by quantifying nuclear staining of pSTAT1 and pSTAT3 in HSV-1 and mock-infected neurons, following direct treatment with IFNγ. IFNγ was chosen as a representative IFN treatment due to its cell-wide effects on STAT phosphorylation and its postulated role as a clinical treatment for peripheral HSV infections (58, 59). As expected, significantly higher levels of pSTAT1 and pSTAT3 were measured in the nucleus of IFNγ treated mock-infected neurons from 2 to 30 h after addition when compared to untreated mock-infected neurons. However, HSV-1 infection reversed this nuclear translocation even in the presence of IFNγ.

HSV-1 has been observed to dampen the IFN response in Vero cell lines by both decreasing the levels of the STAT1 phosphorylation and inhibiting the nuclear translocation of pSTAT1, mediated by the viral protein ICP27 (60). Our results support this data showing both pSTAT1 and pSTAT3 display limited nuclear localization in HSV-1 infected neurons, with or without IFN treatment.

Tyrosine phosphorylation of STAT1 and STAT3 is generally mediated by janus kinase (JAK) proteins, which are activated in response to several cytokines, including type I, II, and III IFNs (61). STAT1 is a transcription factor, and its major role is to establish an antiviral state through transcriptional activation of several genes that interfere with the virus lifecycle (62). Therefore, several viruses have developed evasion mechanisms that aim to inhibit these functions (62). HSV-1 has evolved mechanisms to induce the degradation of STAT1, inhibition of STAT1 phosphorylation, and inhibition of pSTAT1 nuclear translocation (9, 62). However, there are several examples where viruses not only induce phosphorylation of STAT1 but also this phosphorylation of STAT1 plays a positive role in the virus lifecycle. Both influenza A and Sendai viruses induce STAT1 phosphorylation in macrophages (63). Influenza A also induces tyrosine phosphorylation of STAT1 in epithelial cells, where it plays a role in viral RNA synthesis. Inhibition of pSTAT1 reduces influenza A viral load in these cells suggesting that pSTAT1 plays a critical in viral replication (64). Human immunodeficiency virus (HIV) and the closely related simian immunodeficiency virus (SIV) both induce STAT1 phosphorylation, where it plays a role in maintaining the infection, and in the case of HIV, it was independent of IFNγ (65, 66). Inhibition of pSTAT1 in SIV infected macrophages reduces SIV viral load (66). Whereas inhibition of pSTAT1 in HIV infected macrophages results in increased apoptosis of macrophages suggesting that HIV induces pSTAT1 to promote cell survival and maintain macrophages as a persistent HIV reservoir (65).

In addition to IFN, activation of STAT3 is mediated by several cytokine and growth factors including interleukin (IL) -6, IL-10, IL-12, and tumor necrosis factor (67). Previous studies have shown that HSV-1 induces STAT3 phosphorylation in epithelial cells and T cells during early stages of infection (68, 69), and that STAT3 acts as an important transcription factor inducing the expression of HSV-1 ICP0 and TK genes (68). However, STAT3 also has several antiviral effects in animal models regulating the expression of several genes involved in the maintenance of latency in neurons (70). STAT3 knockout mice and cultured cells are also more susceptible to HSV-1 infection (71). To our knowledge, our data are the first to show that STAT1 and STAT3 tyrosine phosphorylation is induced by HSV-1 infection alone (in the absence of exogenous IFN) in neurons. This phosphorylation was localized only to HSV-1 infected neurons. Adjacent uninfected neurons in the same culture were not positive for pSTAT1 or pSTAT3. Further studies will be necessary to determine the mechanisms behind this activation, whether it is through the production of a different cytokine or, more likely, directly due to a viral protein, and whether this activation and inhibition of nuclear pSTAT translocation by all IFN subtypes play a role in promoting the HSV-1 lifecycle in sensory neurons.

To our knowledge, this is the first time that it has been shown that axonal IFN treatment inhibits the release of HSV-1 from the axons of sensory neurons, which highlights the neuro-epidermal junction as a novel intervention site for therapy. We have previously shown that upon HSV-1 infection of the epidermis, DCs such as Langerhans cells and the newly discovered Epi-cDC2s migrate from the epidermis to the dermis, where they undergo apoptosis and transfer HSV-1 antigens to dermal DCs (dDCs). These dDCs migrate to lymph nodes where, in mouse models, they stimulate specific CD4 and CD8^+^ T cells, which are then recruited back to lesions and secrete IFNγ at the neuro-epidermal junction (17, 72, 73). HSV-specific CD8^+^ T cells persist as resident memory T cells at the site of infection for several months after viral clearance. Upon HSV-1 recurrence and epidermal reinfection, they secrete IFNγ triggering an antiviral response in keratinocytes (15, 19, 74). Our data suggest that IFNγ may synergize with local type I IFN production from keratinocytes, Langerhans cells and plasmacytoid DCs, to have additional antiviral actions on the axon termini themselves, limiting the shedding of HSV-1 particles into the epidermis.

Our data also suggest that two potential sites could be explored for therapeutic intervention against recurrent herpes with peripherally applied IFN pathway analogues. (i) Neuronal cell bodies in the ganglia in which axonally applied IFNγ induce an antiviral response and could also inhibit viral reactivation. However, this is counteracted by the effect of HSV-1 infection in limiting pSTAT nuclear translocation. Therefore, this mechanism would need to be elucidated and the pathway blocked. (ii) The neuro-epidermal junction which is already targeted therapeutically by acyclovir. However, this requires uptake into already infected keratinocytes, which may explain its limited effect in treatment and even in prophylaxis where only 48% prevention of transmission to others is achieved (75). Blocking viral exit from epidermal nerves would provide an earlier, more effective strategy particularly as HSV may spread between keratinocytes via interstitial spaces, as well as via cell-to-cell transmission between keratinocytes (76). Such a strategy would require elucidation of the local axonal IFN inhibitory pathways downstream from STAT phosphorylation to choose a suitable agonist/analogue acting directly on the key step in viral exit in the axon terminus. This agonist/analog could also be used prophylactically with ideally greater efficacy than acyclovir or with synergistic effects.

## SUPPLEMENTAL MATERIAL

Fig. S1 mbio.01818-23-s0001.tif Control experiment showing that the cell body and axonal compartments are fluidically distinct and that virus and IFN do not leak between compartments.

Supplemental Figure Legends mbio.01818-23-s0002.docx Legends for Fig. S1 to S5.

Fig. S2 mbio.01818-23-s0003.tif Direct treatment of neurons in the cell body compartment with IFNγ and IFNα-14 does not significantly inhibit HSV-1 release from neurons.

Fig. S3 mbio.01818-23-s0004.tif HSV-1 infection limits the nuclear translocation of pSTAT1 and pSTAT3 even in the presence of IFN.

Fig. S4 mbio.01818-23-s0005.tif Axonal treatment with type I and III IFNs of mock-infected neurons does not induce the nuclear translocation of pSTAT1 and pSTAT3.

Fig. S5 mbio.01818-23-s0006.tif Axonal treatment with type II IFN, but not type I, results in a cell-wide response, at all timepoints tested in mock-infected neurons.

ASM does not own the copyrights to Supplemental Material that may be linked to, or accessed through, an article. The authors have granted ASM a non-exclusive, world-wide license to publish the Supplemental Material files. Please contact the corresponding author directly for reuse.
