# Supplementary figures and images for: Interferon inhibits the release of herpes simplex virus-1 from the axons of sensory neurons
Source: mBio. 2023 Sep 1;14(5):e01818-23. doi: 10.1128/mbio.01818-23 (PMC10653907; doi:10.1128/mbio.01818-23)

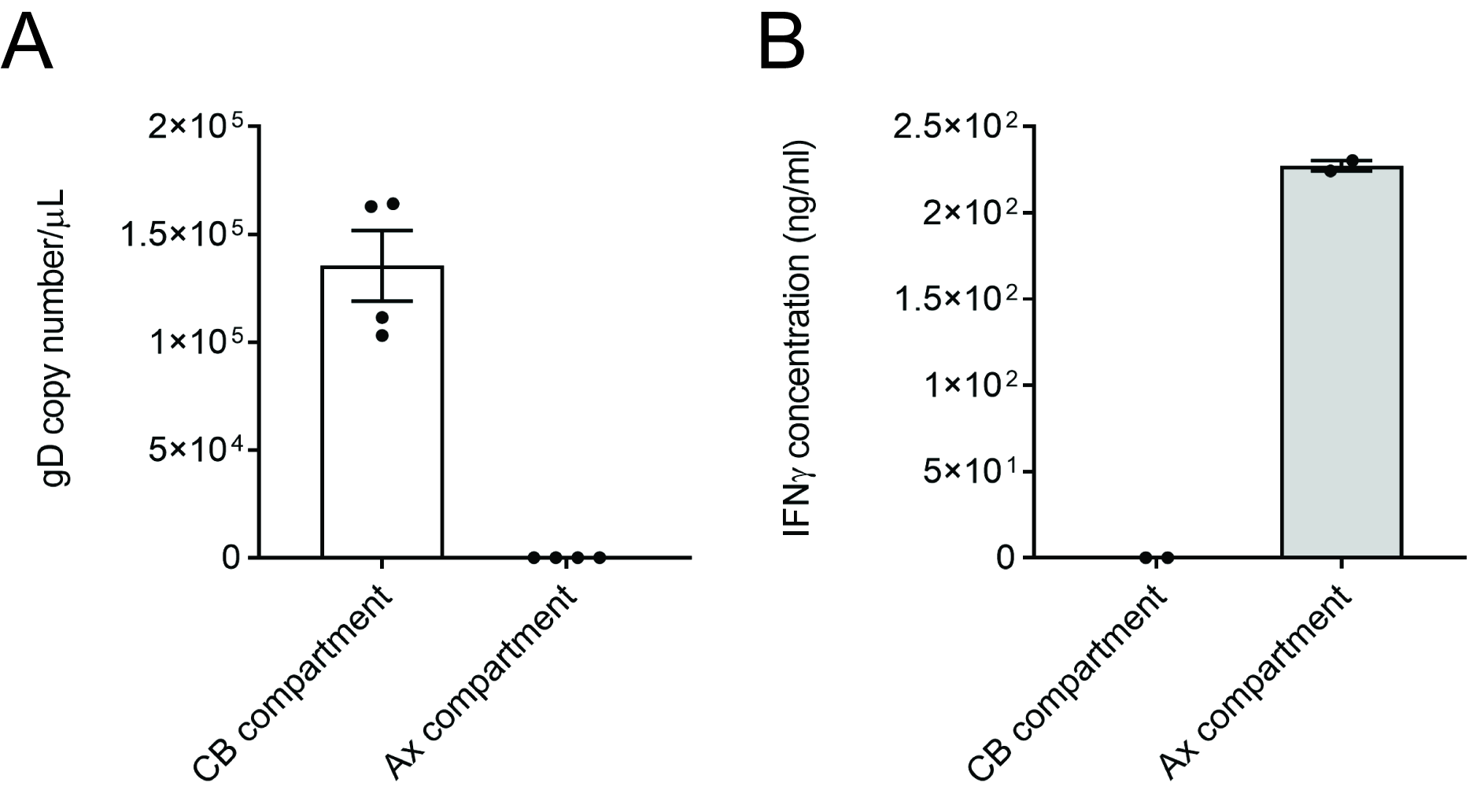

Supplement: Fig. S1 — Control experiment showing that the cell body and axonal compartments are fluidically distinct and that virus and IFN do not leak between compartments. [file mbio.01818-23-s0001.tif]

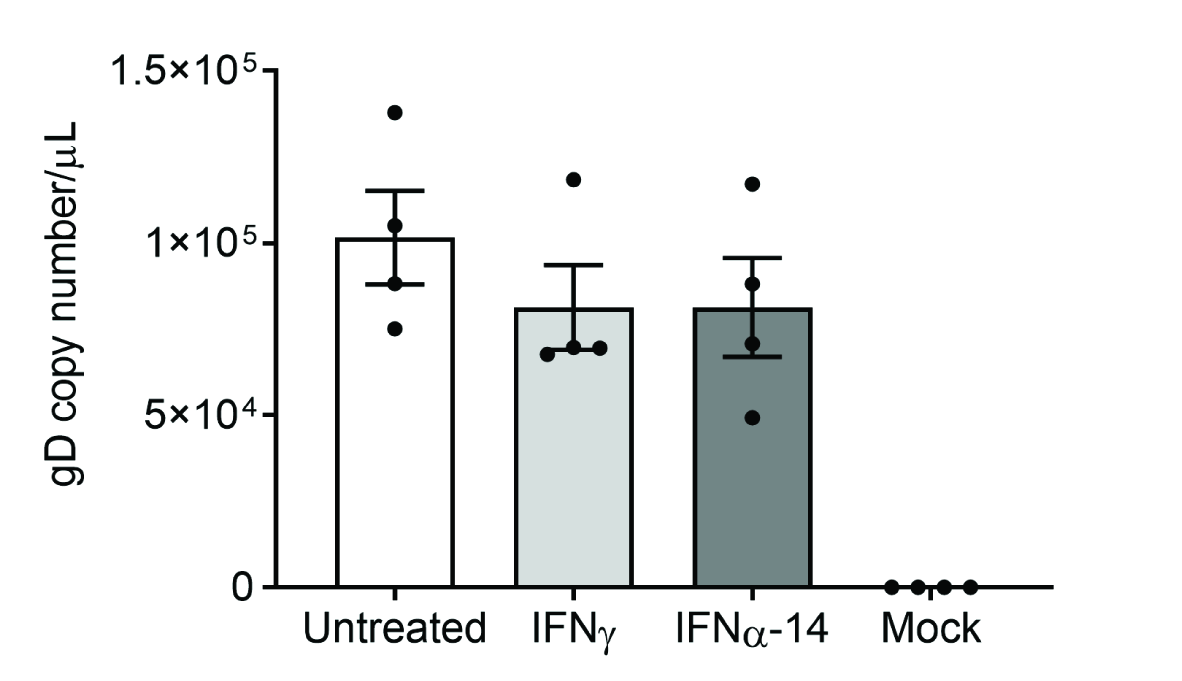

Supplement: Fig. S2 — Direct treatment of neurons in the cell body compartment with IFNγ and IFNα-14 does not significantly inhibit HSV-1 release from neurons. [file mbio.01818-23-s0003.tif]

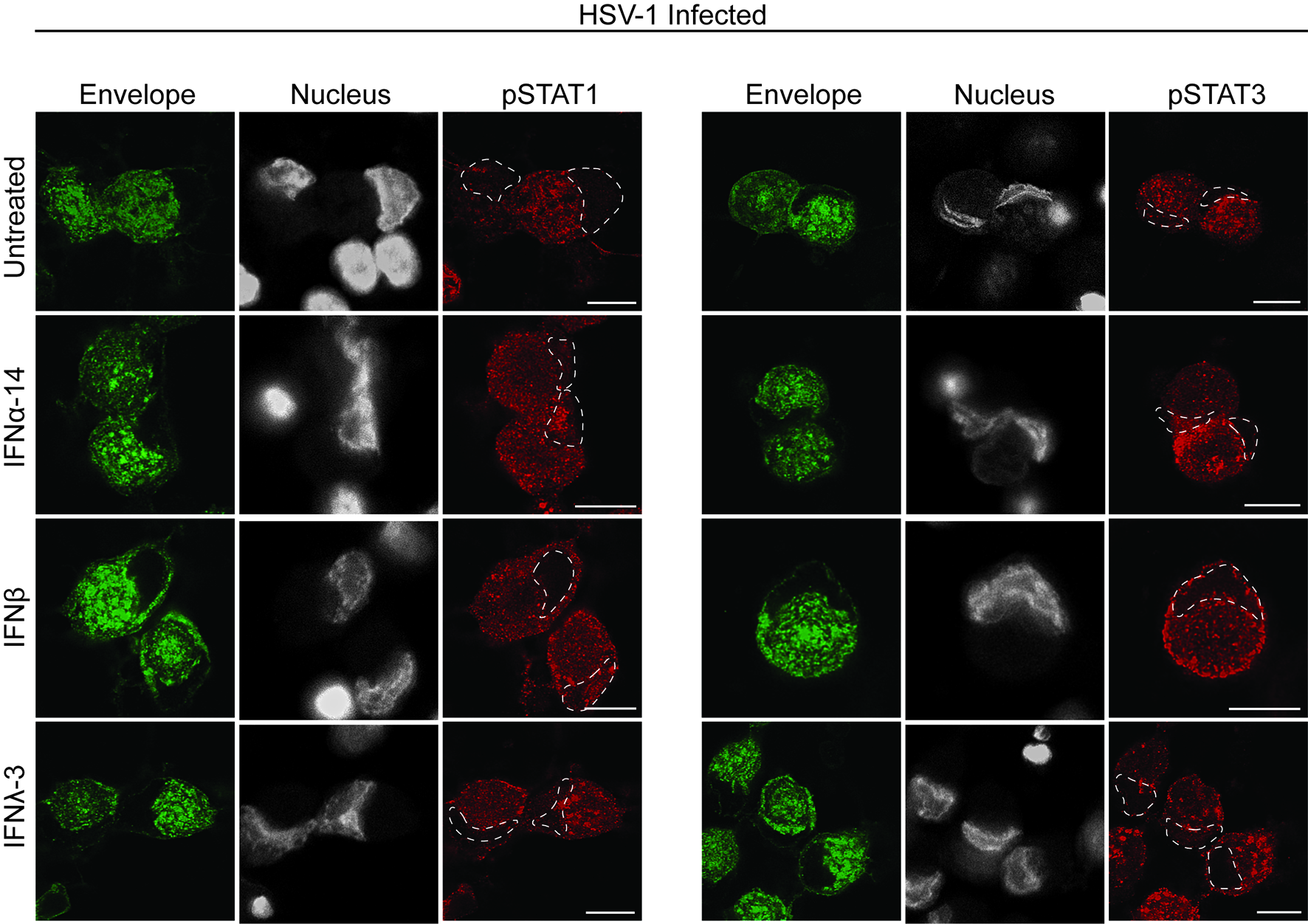

Supplement: Fig. S3 — HSV-1 infection limits the nuclear translocation of pSTAT1 and pSTAT3 even in the presence of IFN. [file mbio.01818-23-s0004.tif]

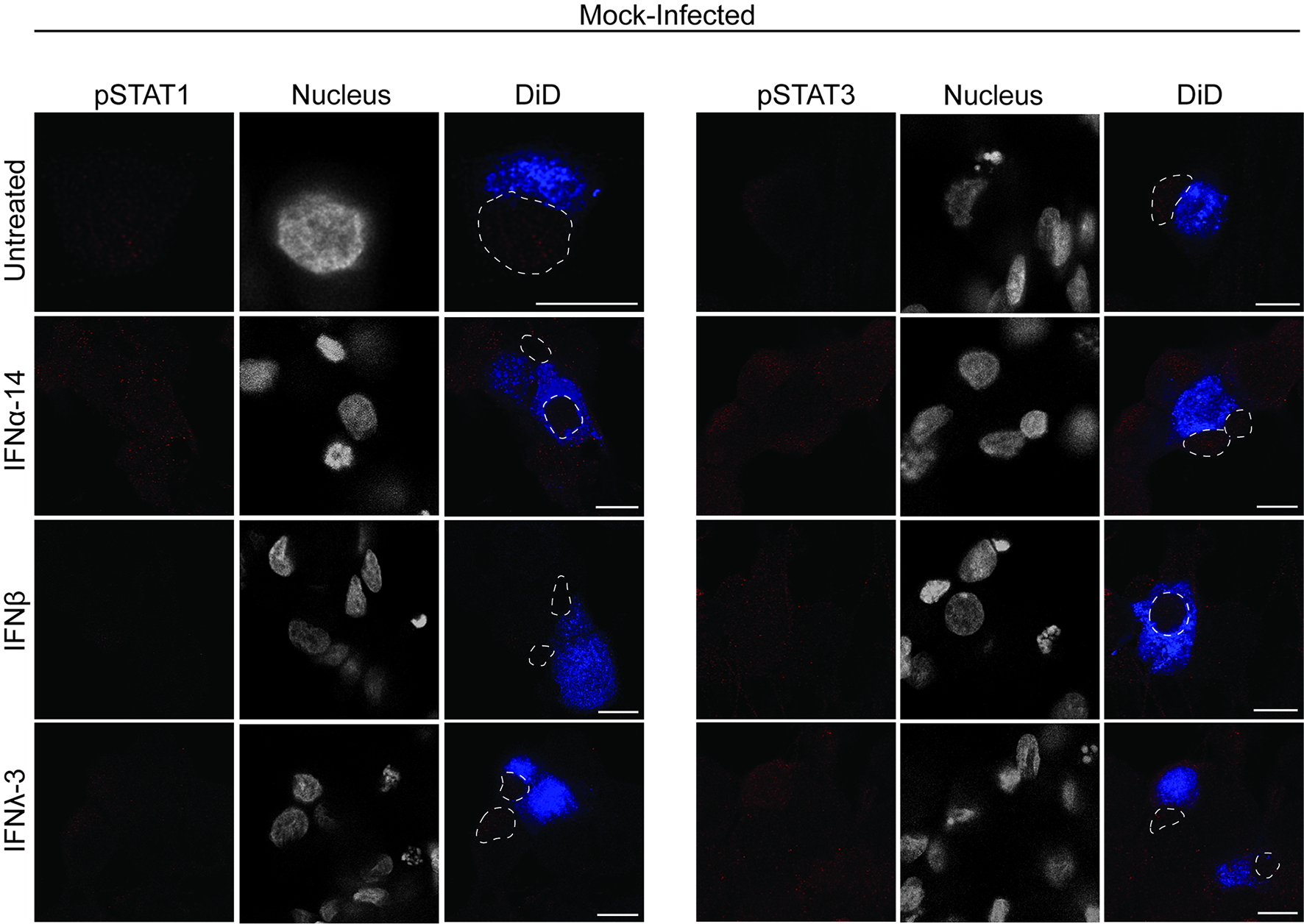

Supplement: Fig. S4 — Axonal treatment with type I and III IFNs of mock-infected neurons does not induce the nuclear translocation of pSTAT1 and pSTAT3. [file mbio.01818-23-s0005.tif]

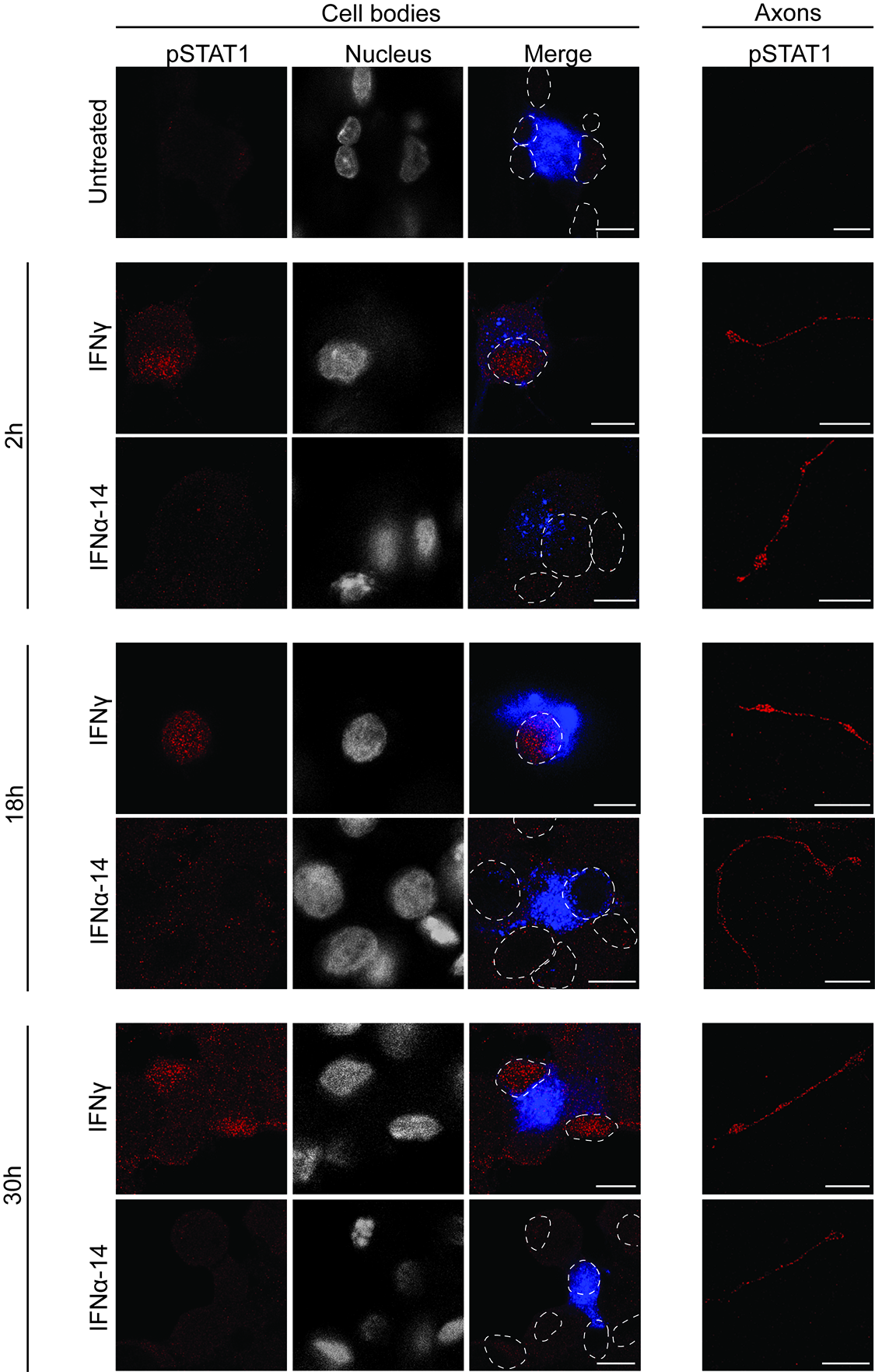

Supplement: Fig. S5 — Axonal treatment with type II IFN, but not type I, results in a cell-wide response, at all timepoints tested in mock-infected neurons. [file mbio.01818-23-s0006.tif]
