# Supplementary material for: Interferon inhibits the release of herpes simplex virus-1 from the axons of sensory neurons
Source: mBio. 2023 Sep 1;14(5):e01818-23. doi: 10.1128/mbio.01818-23 (PMC10653907; doi:10.1128/mbio.01818-23)
Supplement: Supplemental Figure Legends — Legends for Fig. S1 to S5. [file mbio.01818-23-s0002.docx]

**Supplementary Figures**

**S1 Fig. Control experiment showing that the cell body and axonal compartments are fluidically distinct and that virus and IFN do not leak between compartments.** A) HSV-1 was added to the cell body compartment (in the absence of neurons) and incubated at 37 °C. At 30 hpi, the media from both compartments was collected. Viral DNA was extracted from media and analysed by ddPCR targeting the gene encoding for the viral envelope protein gD. n = 4. B) IFNγ was added to axon compartment (in the absence of neurons) and incubated at 37 °C. At 30h, the media from both compartments was collected. An IFNγ ELISA kit was used to measure the concentration of IFNγ in both compartments. n = 2.

**S2 Fig. Direct treatment of neurons in the cell body compartment with IFNγ and IFNα-14 does not significantly inhibit HSV-1 release from neurons.** Neurons in the cell body compartment were directly treated with IFNγ or IFNα-14 for 24h followed by HSV-1 infection. Media from the cell body compartment was collected at 30 hpi. Viral DNA was extracted from media and analysed by ddPCR. n=4.

**S3 Fig. HSV-1 infection limits the nuclear translocation of pSTAT1 and pSTAT3 even in the presence of IFN.** Neuronal cultures were directly treated with IFNα-14, IFNβ, or IFNλ-3 for 24h followed by HSV-1 infection. Cultures were fixed at 30 hpi and immunostained for pSTAT1 (tyr701) or pSTAT3 (tyr705). Cultures were examined using a Leica SP5 II confocal microscope. Micrographs of HSV-1 infected cell bodies showing label for viral envelope protein pUS9 (green), nuclei (grey) and either pSTAT1 (red) and pSTAT3 (red). Dashed lines in the pSTAT1 and pSTAT3 panels represent relevant nuclei. Scale bars = 10 μm. This experiment was performed at the same time as the experiments in Figs 6 and 7A.

**S4 Fig. Axonal treatment with type I and III IFNs of mock-infected neurons does not induce the nuclear translocation of pSTAT1 and pSTAT3.** Axons in the axonal compartment were treated with IFNα-14, IFNβ, or IFNλ-3 as previously described. The lipophilic tracer (DiD) was added to the axonal compartment. Cultures were fixed at 30 hpi and immunostained for either pSTAT1 (tyr701) or pSTAT3 (tyr705). Cultures were examined using a Leica SP5 II confocal microscope. Micrographs of mock-infected neurons showing label for nuclei (grey), lipophilic tracer (blue) and either pSTAT1 (red) or pSTAT3 (red). Dashed lines in the merged panel represent relevant nuclei. Scale bars = 10 μm.

**S5 Fig Axonal treatment with type II IFN, but not type I, results in a cell-wide response, at all timepoints tested in mock-infected neurons.** Axons in the axonal compartment were treated with IFNα-14 or IFNγ as previously described. The lipophilic tracer (DiD) was added to the axonal compartment. Cultures were fixed at 2, 18 and 30 h post-treatment and immunostained for pSTAT1 (tyr701). Cultures were examined using a Leica SP5 II confocal microscope. Micrographs of mock-infected neurons showing label for lipophilic tracer (blue) and pSTAT1 (red) at 2, 18 and 30h post-treatment. Dashed lines represent the nucleus. Scale bars = 10 μm.
